# Implementation Readiness and Adoption of AI-Enabled Voice Electronic Medical Records in Resource-Constrained African Health Systems: Multisite Qualitative Study

**DOI:** 10.2196/100805

**Published:** 2026-09-29

**Authors:** Melika Desalegn, Hocheol Lee, Dawit Wondifraw Tilahun, Alemu Sugi

**Affiliations:** 1 Department of Health Administration Yonsei University Wonju City, Gangwon Republic of Korea; 2 AI Health Data LAB Department of AI Health Informations Management Yonsei University Wonju City, Gangwon Republic of Korea; 3 Department of Radiology St. Paul's Hospital Millennium Medical College Addis Ababa, Addis Ababa Ethiopia; 4 Department of Internal Medicine Care and Cure Medical Center Dar es Salaam, Dar es Salaam United Republic of Tanzania

**Keywords:** artificial intelligence, AI, voice electronic medical records, digital health, multilingual speech recognition, low- and middle-income countries, sociotechnical systems, health information systems

## Abstract

**Background:**

AI-enabled voice electronic medical records (EMRs) are increasingly promoted as tools to reduce clinician documentation burden; however, empirical evidence from multilingual, resource-constrained health systems in sub-Saharan Africa remains limited.

**Objective:**

This study examined health care professionals’ perceptions, anticipated benefits, and concerns regarding AI voice-enabled EMRs in Ethiopia to inform context-sensitive implementation strategies.

**Methods:**

We conducted a qualitative, multisite study using semistructured written responses from 43 Ethiopian health care professionals recruited via purposive and maximum variation sampling between April 11, 2026, and April 21, 2026. Data were analyzed in Taguette using a hybrid deductive-inductive approach integrating 3 complementary frameworks: the Consolidated Framework for Implementation Research (CFIR), the technology acceptance model (TAM), and Normalization Process Theory (NPT). Analytical rigor was strengthened through independent dual coding, structured reconciliation, reflexive memos, and a version-controlled audit trail.

**Results:**

Four overarching themes were identified. First, participants anticipated clear clinical benefits, including reduced typing burden, improved documentation continuity, and enhanced patient interaction, yet expressed substantial concerns about automation errors, accent-related transcription failures, and persistent infrastructural instability. Second, usability barriers, including interface complexity, inadequate training, and digital anxiety, shaped technology acceptance across cadres. Third, participants anticipated shifts in workflow, task distribution, and clinical collaboration as documentation practices evolved. Finally, ethical and governance concerns, particularly regarding data confidentiality, unclear consent procedures, and fear of surveillance, emerged as major determinants of trust. Cross-framework synthesis revealed that adoption readiness was jointly shaped by organizational capacity, usability perceptions, emotional and cognitive responses, and evolving workflow expectations.

**Conclusions:**

Successful implementation of AI voice-enabled EMRs in Ethiopia requires coordinated investments in digital infrastructure, locally adapted language models, strengthened data governance, and iterative user onboarding. These findings underscore the urgency of context-sensitive and ethically grounded approaches when deploying speech-based AI in low-resource health systems.

## Introduction

Health care systems worldwide are undergoing rapid transformation driven by digital health technologies. The World Health Organization’s Global Strategy on Digital Health 2020-2025 recognizes electronic medical records (EMRs) as foundational to achieving universal health coverage [1]. EMRs facilitate structured data storage, retrieval, and communication among health care professionals, improving clinical accuracy and reducing duplication of effort. Recently, AI, particularly voice-based and natural language processing (NLP) technologies, has emerged as a promising complement to conventional EMRs, with the potential to reduce the documentation burden and allow clinicians to focus on patient care.

The integration of AI voice technologies into health care is not without challenges. Key concerns include accurate recognition of diverse accents and multilingual speech, ensuring patient privacy during ambient recording, interoperability with existing EMR platforms, and addressing ethical issues related to data ownership and algorithmic bias [2]. These concerns are amplified in low- and middle-income countries (LMICs), where health information systems are fragmented, digital infrastructure is unreliable, and clinical communication frequently occurs in multiple languages simultaneously.

Although EMR implementation challenges in LMICs are well documented [3,4], little empirical attention has been paid to AI voice-enabled EMR systems, which represent a qualitatively distinct sociotechnical paradigm from typing-based records. Unlike conventional EMRs, AI voice systems rely on real-time speech recognition, NLP, and clinical concept extraction [5]. Ethiopia offers a particularly instructive context for this inquiry. Guided by the Ethiopian Digital Health Blueprint (2020-2024) and the Health Sector Transformation Plan II, the Ministry of Health has made substantial investments in EMR infrastructure across hospitals and health centers. Despite this progress, EMR adoption remains inconsistent, with many facilities operating hybrid paper-digital workflows [3]. Ethiopian clinical communication further complicates adoption: clinicians frequently code-switch among Amharic, *Afaan Oromo*, Tigrinya, English, and local dialects, presenting challenges that most commercial speech recognition systems are ill-equipped to handle.

Existing Ethiopian digital health research has examined EMR usability, digital readiness, and system fragmentation [3,6]; however, no published studies have examined AI-driven voice documentation or its implications for workflow, ethics, and data governance. This study addresses that gap by integrating the Consolidated Framework for Implementation Research (CFIR) [7], the technology acceptance model (TAM) [8], and Normalization Process Theory (NPT) [9] to provide the first multidimensional analysis of how organizational context, individual perceptions, and workflow dynamics shape clinicians’ readiness for AI voice-enabled documentation.

The specific study aims were to generate contextually grounded insights into health care professionals’ perceptions, experiences, and patterns of anticipated use regarding AI-enabled voice EMR systems; to identify infrastructural, organizational, and workforce readiness gaps alongside key facilitators of implementation, with a view to informing national policy and procurement decisions; and to critically examine both enthusiasm and skepticism surrounding AI voice EMRs, elucidating conditions under which these technologies are perceived as trustworthy, usable, and sustainable in routine practice.

## Methods

### Study Design

This was a qualitative, multisite study using semistructured written responses, conducted between April 11, 2026, and April 21, 2026. The analytic approach combined reflexive thematic analysis [10] with a framework matrix informed by CFIR, TAM, and NPT (Figure 1). Reporting followed the COREQ (Consolidated Criteria for Reporting Qualitative Research) checklist [11].

**Figure 1:**
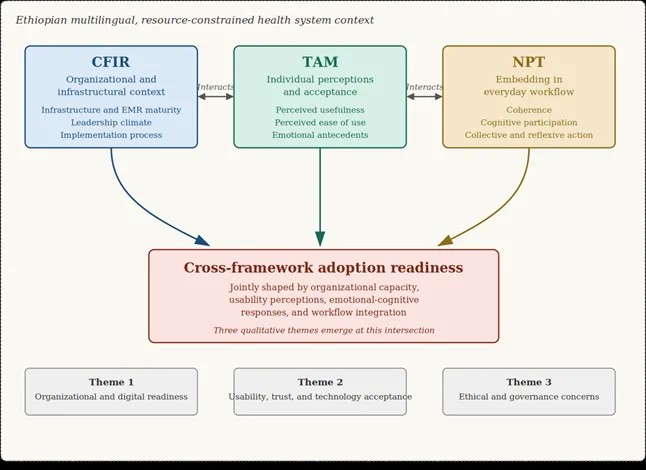
Integrated analytical framework for AI voice-enabled electronic medical record (EMR) adoption. CFIR: Consolidated Framework for Implementation Research; NPT: Normalization Process Theory; TAM: technology acceptance model.

### Study Setting and Sampling

The study was conducted across Ethiopian tertiary hospitals, regional hospitals, and health centers representing varied geographic locations (urban and rural) and EMR maturity levels. Eligible participants included clinicians (general practitioners, residents, nurses, and radiologists), health information officers, IT personnel, facility managers, and public health experts. Purposive sampling, with an intended maximum-variation approach across roles, facility types, and geographic settings, was used via institutional networks to recruit 43 participants. Information power was judged sufficient given the study’s narrow focus on AI voice-enabled EMRs, high participant relevance, strong theoretical scaffolding (CFIR-TAM-NPT), data richness, and iterative analytic approach [12]. The realized sample skewed toward male, younger (aged 20-39 years), and government-facility participants; we therefore characterize the sampling strategy as an approximation of maximum variation rather than a fully achieved one and interpret findings that depend on demographic stratification with corresponding caution. A total of 12 of the 58 (21%) invitees did not complete the questionnaire, and 3 of the 46 (7%) respondents with incomplete core qualitative items were excluded, yielding a final completion rate of approximately 74% (43/58). Detailed role- and facility-level characterization of noncompleting invitees was not systematically captured during data collection, which we note as a limitation.

### Data Collection

Data were collected via an English-language Google Forms (Alphabet Inc) questionnaire containing open-ended items covering participant demographics, EMR experience, awareness and perceptions of AI voice EMRs, and perceived readiness, barriers, and facilitators. Participants completed the form anonymously at their convenience. The instrument was pretested with 3 to 5 comparable participants, and minor revisions were made to improve clarity, item flow, and alignment with the CFIR-TAM-NPT constructs. No standardized prototype, video demonstration, or product description of an AI voice-enabled EMR was provided during data collection; participants therefore responded based on individually constructed mental models of a hypothetical system rather than a shared reference point, a constraint we return to in the Limitations section. Written, semistructured responses via Google Forms were chosen over in-depth interviews or focus groups primarily for feasibility, given the geographic dispersion of participants across tertiary hospitals, regional hospitals, and health centers, and to allow anonymous participation without requiring synchronous scheduling. We recognize that this modality typically yields shorter, less reflexive accounts than spoken interviews, and the resulting data should be interpreted as a distinct qualitative format rather than as equivalent in interpretive depth to interview-based inquiry.

### Data Management

Digital informed consent was obtained within the Google Form prior to participants accessing the qualitative items, following a detailed information sheet covering study purpose, confidentiality, and the right to withdraw. Responses were stored on a secure institutional Google Drive (Alphabet Inc) with restricted access. Deidentified datasets were subsequently exported to encrypted, password-protected local folders for analysis and long-term archiving, consistent with institutional data protection and ethical requirements.

### Data Analysis

All responses were in English and analyzed in Taguette using a hybrid semantic-latent, line-by-line coding strategy. Deductive parent codes were derived from CFIR (inner setting, outer setting, and implementation process), TAM (perceived usefulness and perceived ease of use), and NPT (coherence, cognitive participation, collective action, and reflexive monitoring). Inductive subcodes captured emergent concepts, including multilingual accent mismatch and fear of misinterpretation or surveillance. Two coders independently coded all responses; discrepancies were resolved through side-by-side comparison, reconciliation meetings documented in a decision log, and, when necessary, arbitration by a third reviewer. An audit trail comprising a versioned codebook, reflexive memos, and procedural logs supported transparency.

The integration of CFIR, TAM, and NPT was theoretically motivated: CFIR sensitized the analysis to inner-setting conditions (infrastructure, EMR maturity, and leadership climate); TAM to perceived usefulness, ease of use, and emotional antecedents of adoption; and NPT to the embedding of new documentation routines in everyday practice. Because participants’ accounts of organizational readiness, usability, and workflow integration were substantively interrelated, resulting themes were structured around these overlapping domains of participant experience rather than around single frameworks in isolation; each theme therefore draws on constructs from >1 framework where participants’ own accounts crossed conceptual boundaries.

### Trustworthiness

Trustworthiness was enhanced through methodological and analyst triangulation. Interview findings were compared with contextual documents, including the Ethiopian Digital Health Blueprint and facility EMR guidelines. Independent first-cycle coding by 2 analysts, followed by refinement with a third reviewer, strengthened interpretive credibility. An audit trail, version-controlled codebook, and documented analytic decisions supported dependability. Reflexive memos and peer debriefing enhanced confirmability. Detailed contextual descriptions of the setting, participants, and the Ethiopian digital health context were provided to support transferability.

### Ethical Considerations

Ethics approval was obtained from the institutional review board (IRB) of Yonsei University on April 9, 2026 (1041849-202603-SB-057-02). The study was conducted in accordance with the principles of the Declaration of Helsinki. Participation was voluntary, and participants were informed about the purpose and procedures of the study, the voluntary nature of their participation, their right to decline participation or withdraw, and the intended use of the information they provided. Informed consent was obtained from all participants before participation.

No personally identifiable information was collected. All data were anonymized, securely stored on password-protected and access-restricted drives, and used solely for academic research and health policy purposes. Access to the study data was restricted to authorized members of the research team. Participants received no financial or material compensation for their participation in the study. No photographs, images, or other materials that permitted the identification of individual participants were included in the manuscript.

Given that the study involved no clinical intervention or identifiable patient data and included voluntary, anonymous participation by adult professionals with full rights of withdrawal, ethical oversight was provided by the Yonsei University IRB throughout the study. Retrospectively, ethics approval was also obtained from the IRB of Mekelle University College of Health Sciences–Ayder Comprehensive Specialized Hospital (MU-IRB 2874/2026; expedited approval granted).

### Researcher Positionality

The lead researcher has professional experience in digital health and health systems in Ethiopia, providing contextual depth while simultaneously creating the potential for interpretive bias. To mitigate this, the study incorporated independent dual coding, structured reconciliation procedures, and detailed analytic memos documenting assumptions and decisions. Peer debriefing with colleagues outside the research team further challenged emergent interpretations, ensuring that findings reflected participants’ perspectives rather than the researchers’ a priori expectations.

## Results

### Participant Characteristics

Forty-three health care, IT, and administrative professionals participated, representing diverse roles, educational backgrounds, facility types, and years of experience (Table 1). Most participants were male (n=36, 84%), aged 20 to 39 years (n=37, 86%), and employed in government hospitals (n=34, 79%). Professional roles spanned residents; general practitioners; radiologists; nurses; and a substantial proportion of EMR administrators, IT staff, and public health experts (n=24, 56%). This diversity provided a broad range of perspectives on digital health readiness and EMR familiarity. Participants’ prior exposure to AI-based tools or voice transcription software specifically was not systematically captured in the survey instrument; we note this as a limitation, as baseline familiarity likely shaped how participants interpreted and evaluated the hypothetical system described in this study.

**Table 1:** Participant characteristics (N=43).

Characteristics		Participants, n (%)^a^
Sex		
	Male	36 (84)
	Female	7 (16)
Age group (years)		
	20-29	21 (49)
	30-39	16 (37)
	40-49	3 (7)
	50-59	2 (5)
	≥60	1 (2)
Educational level		
	Master’s degree	15 (35)
	Certificate or diploma	9 (21)
	Residency (in training)	7 (16)
	Subspecialty	5 (12)
	Bachelor’s degree	5 (12)
	PhD	2 (5)
Institution type		
	Government hospital or health center	34 (79)
	Private institution	9 (21)
Professional role		
	Residents	6 (14)
	General practitioners	5 (12)
	Radiologists	5 (12)
	Nurses	3 (7)
	Electronic medical record administrators, IT staff, and public health experts	24 (56)
Professional experience (years)		
	1-5	16 (37)
	6-10	12 (28)
	11-15	8 (19)
	16-20	5 (12)
	>20	2 (5)

^a^Percentages are rounded to the nearest whole number and may not sum to 100% within each variable.

### Theme 1: Organizational and Digital Readiness (CFIR)

Participants consistently identified substantial organizational and infrastructural readiness gaps that shaped their assessments of implementation feasibility. Infrastructure limitations were the most frequently cited barrier: frequent power outages, unreliable internet connectivity, and insufficient hardware, particularly in regional and rural facilities, led many participants to question the viability of cloud-dependent or AI-augmented systems.

#### Anticipated Clinical Benefits

Despite these concerns, participants expressed strong optimism about the potential clinical value of voice-enabled documentation. Reduced administrative burden was the most anticipated benefit, particularly among clinicians managing high outpatient volumes. One participant responded as follows:

Voice documentation is faster and easier for busy clinics. It removes the stress of rushing through notes.

Nurse

Participants also anticipated improvements in documentation accuracy and completeness. Typing was seen as conducive to abbreviations and clinical shortcuts, whereas dictation was perceived to enable richer, more descriptive narratives. One participant responded as follows:

Structured transcribed notes would improve follow-up care. The next clinician would know exactly what happened.

Public health officer

#### Usability Barriers and Skills Gaps

Usability concerns were substantial. Participants described complex or nonintuitive interfaces as sources of anxiety, particularly among less digitally experienced clinicians. Interface navigation barriers were expected to slow adoption and increase the risk of errors. One participant expressed as follows:

Some of us are not used to complicated screens. If the system is not simple, people will give up quickly.

Nurse

Training emerged as a critical theme shaping perceived ease of use. Participants emphasized that digital health tools in Ethiopia were frequently introduced without structured onboarding, phased learning curves, or refresher sessions. The majority advocated repeated, hands-on, iterative training, citing high staff turnover and wide variation in baseline digital skills:

People forget. Training must be repeated until everyone is comfortable.

Senior resident physician

Emotional factors also influenced adoption readiness. Clinicians described anxiety about their confidence, fear of appearing incompetent, and reluctance to seek assistance, particularly among older or less digitally literate staff:

Some colleagues feel shy to ask for help. They pretend to understand but struggle.

Resident

### Theme 2: Usability, Trust, and Technology Acceptance (TAM)

#### Reliability and System Trust

Trust in AI voice-enabled EMRs was highly variable. Although many clinicians associated AI tools with modernization and global innovation, reliability and linguistic accuracy emerged as equally prominent concerns. Fear of misinterpretation, particularly when AI mistranscribed medical terminology or locally accented speech, was pervasive. One participant responded as follows:

If the system misinterprets one word, it may affect patient care.

Physician

Participants also expressed concern about system crashes and operational overdependence in settings with unstable power and network infrastructure.

#### Linguistic Challenges in a Multilingual Context

Linguistic challenges were particularly salient in Ethiopia’s multilingual clinical environment. Clinicians routinely code-switch among Amharic, *Afaan Oromo*, Tigrinya, English, and local dialects; many doubted whether any existing commercial system could accurately process such variation:

Many of us mix Amharic and English when we talk. If the system doesn’t understand both, we will spend more time fixing errors.

Radiologist

This theme demonstrates that perceived usefulness is not determined solely by anticipated efficiency gains but is jointly shaped by historical experience with technology failure, organizational memory of prior EMR rollouts, and expectations of sustained institutional commitment.

### Theme 3: Workflow Integration and Implementation Dynamics (NPT)

#### Workflow Adaptation and Professional Role Boundaries

Participants expected AI voice-enabled EMRs to reduce manual data entry; however, many anticipated unintended tasks shifting as documentation workflows changed. Some predicted redistribution of documentation responsibilities toward nurses or junior staff, potentially blurring established professional boundaries:

If the system writes as we speak, some roles may change. We must be clear about responsibilities.

Public health officer

#### Communication, Collaboration, and Continuity of Care

Participants anticipated that real-time transcription could strengthen interdepartmental communication, reduce duplication of clinical findings, and enhance continuity of care:

Everyone from the lab to the pharmacy can see the same note instantly.

Health information officer

#### Resistance, Change Fatigue, and Institutional Trust

Resistance to new digital tools was common, especially among older staff or clinicians who had experienced prior EMR implementations that were subsequently abandoned or inadequately supported. Past failures had generated skepticism about whether new systems would be maintained long enough to justify the learning investment. One participant responded as follows:

Older staff feel this is for young people; they prefer handwriting.

Administrator

#### Impact on Patient-Provider Interaction

Many clinicians believed that voice documentation could enhance patient engagement by reducing time spent typing during consultations. Speaking while examining was perceived as enabling a more natural and attentive clinical interaction. One participant responded as follows:

Speaking while examining keeps you with the patient, not the computer.

Subspecialist

### Theme 4: Ethical, Governance, and Linguistic Equity Concerns

#### Data Privacy and Informed Consent

Ethical and governance concerns were expressed by nearly two-thirds of participants. Key issues included data confidentiality, uncertainty regarding server location and data ownership, unclear consent procedures, and fear of surveillance or misuse. One participant responded as follows:

If stored on external servers, confidentiality could be compromised.

Health information officer

Participants stressed the need for explicit, multilingual, and culturally appropriate informed consent, noting that audio recording was perceived as inherently more sensitive than typed documentation. Fear of managerial surveillance and concern that voice systems might capture unintended content or be repurposed for performance monitoring were also expressed:

There are no strong laws for digital data here. That makes people anxious.

Physician

Weak national data protection frameworks and unclear accountability structures intensified these concerns, highlighting the need for transparent communication regarding data retention policies, access controls, and audit mechanisms.

#### Linguistic Equity and Accent Bias

Linguistic equity was identified as one of the most significant implementation challenges. Participants feared that accent-related or code-switched speech would produce transcription errors, compromising patient safety and increasing clinician workload. These concerns underscore the ethical importance of developing locally trained models, collecting Ethiopian speech corpora, and ensuring accent-inclusive tuning. Without such measures, voice-enabled EMRs risk reproducing existing digital inequities and further eroding clinician trust.

## Discussion

### Principal Findings

This study provides one of the first in-depth qualitative examinations of health care professionals’ perceptions of AI voice-enabled EMRs in Ethiopia. Four overarching findings emerged. First, clinicians expressed genuine optimism about the potential clinical and operational benefits of voice documentation, framing efficiency gains as directly linked to care quality and patient safety rather than mere administrative convenience. Second, substantial usability, trust, and linguistic barriers constrain readiness for adoption, and these barriers are deeply embedded in Ethiopia’s broader digital health landscape. Third, participants anticipated that voice-enabled documentation would reshape workflow patterns and professional role boundaries, requiring deliberate management of task redistribution and interdepartmental coordination. Fourth, ethical and governance concerns, particularly regarding data confidentiality and linguistic equity, represent critical determinants of trust that implementation strategies must proactively address.

### Perceived Usefulness and Clinical Value

Participants’ strong expectations that AI voice-enabled EMRs could reduce documentation burden and improve workflow efficiency mirror emerging evidence from LMIC digital health studies. Research has demonstrated that digital documentation tools in resource-constrained settings substantially reduce administrative load and increase perceived productivity [4], findings closely paralleling clinicians’ views in this study. Our participants described improved documentation not merely as an efficiency gain but as an enabler of clinical adequacy, particularly relevant in Ethiopia’s high-volume outpatient settings, where staffing is limited and caseloads are large.

These findings are consistent with global reviews of speech-based AI in health care, which demonstrate that voice interfaces reduce task switching, manual data entry, and cognitive overload, thereby supporting clinical focus [5]. Participants also believed that voice-enabled systems could improve documentation accuracy and completeness: typing was seen as conducive to abbreviations and shortcuts, whereas dictation was perceived to allow richer clinical narratives. This aligns with evidence from high-income settings suggesting that speech-based documentation can improve continuity of care and interdepartmental communication [12], although this association has not yet been empirically tested in the Ethiopian context and is offered here as a plausible parallel rather than a confirmed finding.

### Usability, Skills, and Trust Barriers

Despite their optimism, participants expressed significant concerns about usability and digital readiness. Interface complexity, unfamiliar navigation pathways, and fear of error emerged as major barriers, echoing global literature, largely drawn from high-income settings, emphasizing the importance of intuitive interfaces for speech AI systems in clinical environments [12]; the extent to which these findings are transferable to Ethiopia’s clinical environments remains to be empirically established. In settings where digital experience is uneven, even moderate interface complexity can produce cognitive overload and impede adoption. These findings align with recent systematic work on human-centered AI agent design in health care and education, which emphasizes that trust and usability are shaped as much by interface transparency and adaptive onboarding as by underlying model performance [13], suggesting that implementation recommendations for AI voice EMRs in Ethiopia should draw explicitly on human-centered design principles rather than treating usability as a secondary concern to technical accuracy.

Training deficits were among the strongest barriers identified. Clinicians repeatedly described the need for structured, phased, and iterative training, reflecting LMIC research demonstrating that digital health tools commonly fail when training is superficial or delivered as a 1-time event [4]. In Ethiopia’s context of wide variation in digital literacy, training is not simply a capacity-building mechanism but an equity imperative enabling all clinical cadres to participate meaningfully in digital transformation.

Emotional and trust-related barriers further shaped usability perceptions. Clinicians described shame, embarrassment, and reluctance to seek help, particularly among older or less digitally literate staff. These findings echo research demonstrating that emotional responses such as anxiety and hesitation strongly influence automation adoption in clinical settings [2]. In Ethiopia, these emotions are amplified by prior experiences of EMR failure, which have generated skepticism about system longevity and institutional commitment. Because the sample was heavily concentrated among younger clinicians (37/43, 86%; aged 20-39 years; Table 1), with older clinicians who are described here as experiencing greater digital anxiety substantially underrepresented, this finding should be interpreted as suggestive rather than representative of age-related variation in digital anxiety across the Ethiopian health workforce more broadly.

Concerns about reliability and the risk of clinical errors from mistranscription were also prominent. The fear of system crashes in facilities with unstable power and connectivity raised doubts about operational feasibility. Studies in African and Asian settings similarly report that mistranscription of non-Western accents and medical terminology remains a persistent challenge for automated speech recognition (ASR) technologies [14]. Ethiopia’s multilingual clinical communication, frequently involving code-switching among Amharic, *Afaan Oromo*, Tigrinya, and English, presents unique challenges that current commercial AI systems are not equipped to handle. These concerns, while theoretically plausible and consistent with the broader literature, are based on participants’ anticipated rather than observed transcription failures, since no live ASR testing on Ethiopian languages was conducted in this study. A recent non–peer-reviewed letter to the editor proposing retrieval-grounded evaluation of conversational AI in clinical risk-assessment contexts offers a useful conceptual template, rather than empirical precedent, for testing these anticipated failure modes in future research [15]. These linguistic risks nonetheless constitute a major trust barrier that future implementation must explicitly address.

### Workflow Integration and Labor Dynamics

Participants described how AI voice-enabled EMRs could reshape workflow patterns, labor distribution, and clinical coordination. Many anticipated improvements in patient flow, reduced documentation backlogs, and faster consultation turnaround, consistent with evidence that speech-based tools reduce administrative delays [5]. However, concerns about unintended task shifting were equally prominent: clinicians worried that nurses or junior staff might inadvertently become default scribes, a dynamic observed in LMIC digital health projects where technology introduction unintentionally redistributes documentation labor [16]. Explicitly clarifying role expectations and monitoring labor equity are therefore essential preconditions for ethical implementation.

Participants also highlighted the potential of real-time transcription to improve interdepartmental communication, continuity of care, and reduce the duplication of clinical findings, consistent with global EMR research demonstrating the value of interoperable documentation [17]. However, these benefits depend heavily on system interoperability. Ethiopia’s current digital landscape is characterized by hybrid paper-digital workflows, uneven EMR adoption, and limited system integration. Participants warned that adopting AI voice tools without equivalent investment in interoperability could worsen existing digital fragmentation.

The impact of voice documentation on patient-provider interaction was also discussed. Many clinicians felt that voice interfaces could reduce typing distractions and restore clinical presence, echoing findings reported from high-income settings [12]; however, this contextual transfer is not empirically confirmed for Ethiopia and should be treated as a plausible hypothesis rather than an established parallel, given substantial differences in infrastructure, patient volume, and clinical workflow. However, participants recognized that high error rates or frequent need for correction could paradoxically increase cognitive burden and interrupt consultations, a tension noted in the global literature [17]. Resistance to change, particularly among staff who had experienced failed digital implementations, further shaped workflow expectations, underscoring the importance of stable infrastructure and sustained institutional support [18].

### Ethical, Governance, and Linguistic Equity Considerations

Ethical and governance concerns emerged as major cross-cutting findings. Clinicians expressed uncertainty about where voice data would be stored, who would control it, and whether external server involvement would compromise confidentiality. These concerns mirror global AI governance debates emphasizing data sovereignty, transparency, and accountability [1]. In Ethiopia, where national data protection regulations are still evolving, unclear governance structures amplify fears of vulnerability to misuse, unauthorized secondary use, or data breaches. Recent work on institutional AI governance argues that external regulatory frameworks alone are insufficient and must be complemented by internal, organization-level governance mechanisms such as vendor accountability structures and internal audit capacity [19]; this distinction is particularly salient in the Ethiopian context, where institutional governance capacity may need to substitute for, rather than simply await, external regulatory maturity.

Consent emerged as a particularly sensitive dimension. Participants stressed that voice recordings were perceived as inherently more intrusive than typed data, necessitating explicit, multilingual, and culturally appropriate consent processes. This aligns with global ethical frameworks emphasizing meaningful, context-sensitive consent in AI-mediated clinical interactions [2]. Fear of managerial surveillance and concern that voice systems might capture unintended content or be repurposed for performance evaluation were also expressed, underscoring the need for transparent data boundary policies, retention controls, and audit mechanisms.

Linguistic equity represents perhaps the most distinctive implementation challenge in Ethiopia’s context. Participants consistently anticipated that commercial ASR systems would misrecognize their accents and code-switched speech, a concern broadly consistent with emerging evidence drawn mainly from preprint studies of general-purpose ASR performance in African and other underresourced-language settings, suggesting that commercial systems can disproportionately misrecognize non-Western accents and languages with limited training data [14,20]. This literature has not yet directly evaluated Ethiopian languages or Ethiopian-accented clinical speech, so whether these patterns hold in Ethiopia specifically remains an empirical question rather than an established finding. Clinicians’ concerns about accent-based errors and code-switched speech nonetheless highlight the ethical imperative of developing locally trained models, collecting Ethiopian speech corpora, and ensuring accent-inclusive system tuning.

Without such investments, voice-enabled EMRs risk reproducing and deepening existing global digital inequities.

### Strengths and Limitations

This study is among the first qualitative inquiries into AI voice-enabled EMR adoption in Ethiopia and provides context-specific insights into an underresearched area. The multiframework approach (CFIR-TAM-NPT) offered a comprehensive lens encompassing infrastructure readiness, usability, and workflow integration. Participant diversity across roles, departments, and facility types enhanced the richness and credibility of the findings. Rigorous qualitative procedures, including independent dual coding, structured reconciliation, and thematic mapping, supported dependability and trustworthiness.

Several limitations warrant consideration. First, the study did not involve real-time testing of an operational AI voice-enabled EMR; participants’ responses were based on anticipatory perceptions rather than direct experience, potentially introducing perceptual bias in both directions (overestimating risks such as surveillance or overemphasizing expected efficiency gains). Second, patient and caregiver perspectives were not included; future research should examine how voice-based documentation and ambient audio capture are experienced by those receiving care. Third, and most consequentially given this study’s central focus on linguistic equity, requiring exclusively English-language responses created a direct tension with the study’s own themes: participants who routinely code-switch among Amharic, *Afaan Oromo*, Tigrinya, and English in clinical practice were required to respond in English alone, likely introducing self-selection bias that systematically underrepresents the clinicians most affected by accent-related ASR failure, namely those with lower English proficiency working in lower-resource regional facilities. A follow-up study incorporating multilingual data collection would be needed to represent these voices directly. Fourth, ethics approval from the Mekelle University IRB (MU-IRB 2874/2026; expedited approval granted September 4, 2026) was obtained retrospectively, after data collection had concluded, rather than prior to recruitment; although ethical oversight for the study was maintained throughout via the Yonsei University IRB, we acknowledge that prospective dual-country review is preferable and recommend it as standard practice for future studies recruiting Ethiopian health professionals at scale. Fifth, because this study was conducted in a single country, any transferability of these findings to other African health systems is analytic rather than statistical; readers should treat cross-national relevance as conceptual guidance to be tested locally rather than as a representative generalization across the region. Finally, the cross-sectional design limits the ability to capture longitudinal adoption dynamics.

### Implications for Policy, Practice, and Research

The recommendations below are derived from participants’ anticipatory perceptions of a hypothetical system rather than from evidence of an operational deployment and should accordingly be read as priorities for context-sensitive piloting and empirical validation rather than as directly evidenced operational mandates. These findings underscore the need for a coordinated national strategy for AI-enabled documentation within Ethiopia’s broader digital health agenda. Policymakers should prioritize strengthening digital infrastructure, standardizing EMR maturity across facility levels, and ensuring interoperability before any large-scale deployment of voice-based tools, with these priorities tested through phased, evaluated pilots rather than implemented at scale based on this study alone. Ethiopia’s emerging data protection frameworks would likely benefit from expansion to include explicit provisions for voice data governance, algorithmic transparency, and vendor accountability, although the specific form this should take requires further empirical and legal analysis beyond the scope of this study. The development of national Ethiopian language speech corpora and locally adapted speech recognition models is suggested here as a strategic public-good investment worth piloting to mitigate linguistic inequities and improve contextually appropriate AI performance.

For health facilities and implementing partners, the findings highlight the importance of embedding AI voice-enabled EMRs into existing workflows rather than overlaying them onto fragile systems, a principle that should be tested and refined through phased implementation rather than assumed. Effective adoption likely requires phased rollouts, continuous hands-on training, and accessible troubleshooting mechanisms that accommodate diverse digital competency levels, although the specific design of these mechanisms should be informed by piloting rather than derived directly from this study’s anticipatory data. Clear communication about data boundaries, consent procedures, and intended system use appears important for building trust and reducing fear of surveillance based on participants’ expressed concerns. Facilities should consider proactively managing potential labor redistribution by establishing explicit role expectations and monitoring the equity impact of AI documentation tools on nursing and junior clinical staff, as this concern was raised by participants but not observed directly.

Future research should prioritize real-world evaluation of ASR accuracy across Ethiopian languages, dialects, and multilingual code-switching. Longitudinal implementation research is needed to examine adoption trajectories, workflow changes, and organizational resilience in hybrid paper-digital contexts. Mixed methods and controlled studies can assess the clinical impact of voice-based documentation on decision-making, patient safety, and care continuity. Finally, comparative research across African and multilingual health care systems will advance global understanding of linguistic equity, data sovereignty, and ethical governance in AI-mediated clinical environments.

### Conclusions

This study provides one of the first comprehensive qualitative examinations of how health care professionals in Ethiopia perceive the feasibility, value, and risks of AI voice-enabled EMRs. Across diverse clinical cadres and facility types, participants expressed genuine optimism about the potential of voice documentation to reduce administrative burden, improve workflow efficiency, and strengthen continuity of care, benefits that are particularly salient in high-volume, resource-constrained environments. At the same time, participants identified substantial usability challenges, linguistic limitations, infrastructural fragility, and ethical concerns that substantially constrain adoption readiness.

Integrating CFIR, TAM, and NPT demonstrates that successful implementation depends not only on technological performance but equally on organizational maturity, digital literacy, workflow integration, and trust in governance systems. Participants’ reflections reveal that AI voice-enabled EMRs are experienced as sociotechnical innovations: their value is coproduced through interactions among infrastructure conditions, emotional and cognitive responses, professional identities, and the wider policy environment.

These findings highlight the promise and precarity of speech-based AI in LMIC health systems. Although clinicians recognize significant clinical and operational advantages, this technology will not succeed without sustained investment in local language adaptation, robust data protection frameworks, iterative training, and interoperable digital infrastructure. Strengthening these foundations is essential to ensure that AI-enabled voice EMRs enhance, rather than undermine, equity, safety, and trust in Ethiopia’s digital health transformation.

## Abbreviations

ASR: automated speech recognition
CFIR: Consolidated Framework for Implementation Research
COREQ: Consolidated Criteria for Reporting Qualitative Research
EMR: electronic medical record
IRB: institutional review board
LMIC: low- and middle-income country
NLP: natural language processing
NPT: Normalization Process Theory
TAM: technology acceptance model

## Data Availability

The datasets generated and analyzed during this study are not publicly available because they contain potentially identifiable and sensitive information obtained from qualitative interviews with health care professionals. Deidentified data may be made available by the corresponding author upon reasonable request, subject to ethical and institutional requirements and approval.
